# Structure-function relationships in ABCG2: insights from molecular dynamics simulations and molecular docking studies

**DOI:** 10.1038/s41598-017-15452-z

**Published:** 2017-11-14

**Authors:** Ricardo J. Ferreira, Cátia A. Bonito, M. Natália D. S. Cordeiro, Maria-José U. Ferreira, Daniel J. V. A. dos Santos

**Affiliations:** 1 Research Institute for Medicines (iMed.ULisboa), Faculty of Pharmacy, Universidade de Lisboa, Av. Prof. Gama Pinto, 1649–003 Lisboa, Portugal; 2 LAQV@REQUIMTE, Department of Chemistry & Biochemistry, Faculty of Sciences, University of Porto, Rua do Campo Alegre, 4169–007 Porto, Portugal

## Abstract

Efflux pumps of the ATP-binding cassette transporters superfamily (ABC transporters) are frequently involved in the multidrug-resistance (MDR) phenomenon in cancer cells. Herein, we describe a new atomistic model for the MDR-related *ABCG2* efflux pump, also named breast cancer resistance protein (BCRP), based on the recently published crystallographic structure of the *ABCG5/G8* heterodimer sterol transporter, a member of the ABCG family involved in cholesterol homeostasis. By means of molecular dynamics simulations and molecular docking, a far-reaching characterization of the *ABCG2* homodimer was obtained. The role of important residues and motifs in the structural stability of the transporter was comprehensively studied and was found to be in good agreement with the available experimental data published in literature. Moreover, structural motifs potentially involved in signal transmission were identified, along with two symmetrical drug-binding sites that are herein described for the first time, in a rational attempt to better understand how drug binding and recognition occurs in *ABCG2* homodimeric transporters.

## Introduction

Multidrug resistance (MDR) to anticancer agents is a major health concern worldwide due to the long-lasting physical and psychological outcomes^1^. Herein, MDR is a multi-factorial phenomenon often connected to over-expression of ATP Binding Cassette (ABC) transporters at the cell surface, acting by decreasing the intracellular accumulation of cytotoxic drugs and impairing the success of chemotherapeutic regimens^2^. Of the 48 members of the human ABC family, P-glycoprotein (P-gp, *ABCB1*)^3^, Multidrug-Resistance Protein 1 (MRP1, *ABCC1*)^4^ and Breast Cancer Resistance Protein (BCRP, *ABCG2*)^5–7^ are the most important transporters in cancer MDR, although a new member of the ABCB family (*ABCB5*)^8,9^ is also gaining relevance in melanoma resistance to anticancer agents.

*ABCG2* in MDR was identified almost simultaneously by several groups in MCF-7/AdrVp cell lines (BCRP or MXR)^5,7^ and in placenta (ABCP)^6^. In both MCF-7/AdrVp3000 (over-expressing *ABCG2*) and full length *ABCG2* cDNA-transfected MCF-7 breast cancer cells, the over-expression of this transporter conferred resistance to several xenobiotics including mitoxantrone, doxorubicin and daunorubicin, also reducing the intracellular accumulation of Rhodamine-123 (R123) by an ATP-dependent mechanism^5,7^. Although studied due to its important role in MDR, *ABCG2* also participates in normal detoxification mechanisms that can be found in previoulsy published literature^10–12^.

**Figure 1:**
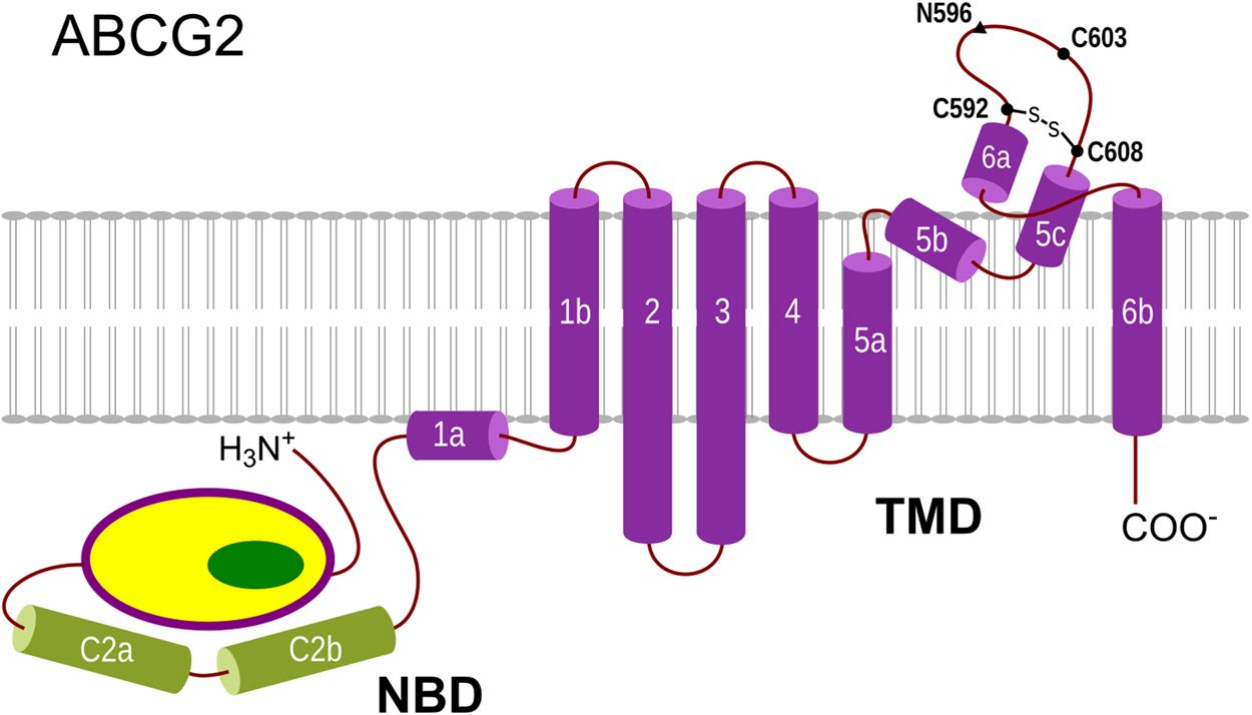
Membrane topology model for *ABCG2*. Cysteine residues involved in disulfide bridges are depicted as circles and *N*-glycosilated residue as a triangle. The *de novo* intracellular C2 helices are depicted in green. TMD, transmembrane domain; NBD, nucleotide-binding domain (adapted from Taylor *et al*.)^18^.

*ABCG2* is characterized as a “half-transporter”, hypothetically comprising a transmembrane domain (TMD) with 6 α-helices that spawns the membrane bilayer and a nucleotide-binding domain (NBD) where ATP binds and hydrolyzes. However, unlike P-gp that shows a TMD-NBD arrangement in both halves, *ABCG2* shows a distinct domain organization where the NBD precedes the TM domain (in a NBD-TMD arrangement, Fig. 1)^13^. Moreover, *ABCG2* includes a second canonical ABC signature (positions 352–356, LSGGE) also coupled with ATP binding and/or hydrolysis but not related with substrate specificity^14^. Although higher orders of oligomerization were also described, namely tetrameric and dodecameric forms, to become a fully functional transporter *ABCG2* forms a homodimer^15,16^. Furthermore, with only a minority dimer population being registered, it was recently determined that tetrameric complexes are the most predominant ones in plasma membranes^17^. The possibility that such higher oligomeric forms may serve as a regulator for functional dimeric *ABCG2* transporters^13^ has become increasingly accepted.

The role of specific cysteines in *ABCG2* oligomerization has also been thoroughly studied since the discovery of *ABCG2* homodimers. While an early study by Kage *et al*. claimed that *ABCG2* homodimerization was due to the formation of an intermolecular disulfide bond at position 603^15^, it was later concluded that such disulfide bond is not essential for protein expression and function^13,16,19–21^. Although present in the absence of reducing agents^15^, and as the dimeric *ABCG2* was found only in isolated membranes, the presence of a -S-S- bridge can also derive from oxidation processes during cell lysis and membrane preparation^13^. Furthermore, it was also shown that only 35% of *ABCG2* was cross-linked as dimers^13^ and that non-covalent protein-protein interactions between transmembrane helices 5 and 6 (TM5-loop-TM6) could also be involved in *ABCG2* oligomerization^22^.

Similarly, the role of an intramolecular disulfide bond between residues 592 and 608 was initially also thought to be crucial for the activity of the transporter^20^. However, while in one study *ABCG2* variants without cysteines in the extracellular loop (C592, C603 and C608) showed similar cellular location and drug-stimulated activity comparable to the wild-type (WT) protein^23^, other two studies concluded that i) protein trafficking to the plasma membrane may be impaired in the presence of C592A/C603A or C603A/C608A mutations but not C592A/C608A^20^ and (ii) the intramolecular disulfide bond is an important “checkpoint” that determines the fate of *de novo* synthesized *ABCG2* proteins^24^. Therefore, it seems that further studies are needed to fully understand the role of inter- and intramolecular disulfide bonds in *ABCG2* expression and function.

Regarding *ABCG2* function, it was shown that mutation of an arginine by a threonine or glycine residue at position 482 increased efflux function towards R123 and anthracyclines as daunorubicin^16^, not interfering directly with substrate binding but affecting other important factors as ATP-related energy coupling, which is intimately linked to the conformational changes, and/or signal transduction mechanisms^25,26^. As for P-gp, the mutation of the aspartate residue in Walker B motif (Mg^2+^-chelating motif, D210N) led to a non-functional protein that was still able to be expressed at the cell surface. Moreover, as it was also found that *ABCG2* prefers detergent-resistant cell membranes characterized by high cholesterol content, cholesterol depletion induces a marked decrease on *ABCG2* transport activity without affecting the localization of the transporter within the cell membrane^27^. Interestingly, 30 molar-% cholesterol content was found to provide the best conditions for optimal *ABCG2* ATPase activity. As such, cholesterol is now considered an essential activator of *ABCG2* function, probably acting as a slowly transported substrate, an allosteric co-activator or a co-transporter^28^. Indeed, at least five cholesterol recognition/interaction amino acid consensus (CRAC)^29^ were identified in the *ABCG2* transporter, but while mutation in Y413 and Y570 induced higher levels of ATPase activity that were not dependent of cholesterol presence, only one (Y413) was able to modulate ATPase activity in the presence of cholesterol^30^.

To date, P-gp is the most studied ABC transporter involved in MDR. Since its publication in 2009^31^, numerous structure-based studies allowed a deeper understanding of the molecular mechanisms involved not only in drug binding and recognition^32–35^ but also about conformational changes intimately related with the efflux mechanism itself^36–39^. Quite recently, the publication of the first structural models of the bovine Multidrug Resistance Protein 1 (MRP1, *ABCC1;* PDB ID: 5UJA)^40^ and human Breast Cancer Resistance Protein (BCRP, *ABCG2*; PDB ID: 5NJ3)^18^ provided new templates for novel structure-based studies, aiming for the development of novel MDR inhibitors but also for studies concerning a greater comprehension about the efflux mechanism in these ABC transporters.

Preceding the publication of the human *ABCG2* structure, the structure of a heterodimeric sterol transporter *ABCG5/G8* ^41^ revealed a new transmembrane organization that was thought to be characteristic of the ABCG family. Indeed, while sharing low amino acid identity (28%), *ABCG5* and *ABCG8* revealed a high degree of structural conservation with a relatively low root mean square deviation (RMSD = 2.0 Å) between both halves^41^. Likewise, *ABCG5* and *ABCG8* share similar identities (27% and 26% respectively) and similarities (48% and 44% respectively) with the newly published *ABCG2* structure, thus being a suitable template for studying not only the dynamics of *ABCG5/G8* heterodimer but also, by homology modeling, the *ABCG2* homodimer. This hypothesis is supported by the fact that i) despite their low identities and similarities, the structural conservation between *ABCG5* and *ABCG2* monomers remains high (RMSD = 2.4 Å) while between *ABCG8* and *ABCG2* is lower (RMSD = 3.8 Å), ii) in the original *ABCG5/G8* publication a homology model of the *Drosophila* white/brown heterodimer was obtained following a CLANS network analysis showing that the TMDs of both transporters shared a high pairwise similarity relationship based on their FASTA sequences and iii) the same pairwise relationship can be observed between *ABCG5/G8* and *ABCG2* ^41^. To that matter, computational approaches as molecular dynamics (MD) and docking are valuable tools that can be used, in a similar approach as for P-gp^36^, to further refine a homology model of the *ABCG2* homodimer and to evaluate the structural dynamics of the *ABCG2* transporter, also unveiling new information on hypothetical drug-binding locations that can be used to better understand the *ABCG2* role on MDR.

## Results

### ABCG2 model validation

The publication of the *ABCG5/G8* crystallographic structure showed that despite the low sequence identity between *ABCG5* and *ABCG8* (28% amino acid identity), a high degree of structural conservation could still be found^41^. Thus, as *ABCG5* and *ABCG2* share 27% amino acid identity, a high pairwise similarity between both FASTA sequences and a high degree of structural conservation (as seen by their RMSD), the homodimeric *ABCG5* transporter is a more suitable template for *ABCG2*. After obtaining the full-length *ABCG2* homology model, structure validation servers were used to assess the quality of the model (Table 1). Furthermore, an evaluation of the recently published human *ABCG2* cryo-EM structure^18^ was also performed to compare and validate the herein developed *ABCG2* model.

**Table 1:** Structural validation of the full-length *ABCG2* models.

		Homology modeling							
		Errat	MolProbity		SwissModel			PROCHECK	
Models		Score	Score	Percentil	QMEAN6^45^	Z-Score^43^	DFIRE^46^	Morris^47^	G-factors^48^
Crystal	G5G8	78.891	3.44	10^th^	0.547	−2.187	−1823.68	1-2-3	−0.22
	*ABCG2* ^a^	76.539	1.91	80^th^	0.498	−2.97	−1359.71	1-1-2	0.03
Homology	*ABCG2* ^b^	67.820	3.5	9^th^	0.526	−2.468	−1645.97	1-1-3	−0.28
	G5_DIMER_	78.075	3.52	8^th^	0.564	−1.996	−1812.30	1-2-3	−0.18
	G2_MODEL_	85.714	1.73	88^th^	0.444	−3.53	−1811.41	1-2-3	−0.18
	G2_FULL_	86.356	1.73	88^th^	0.441	−3.297	−1797.77	1-2-3	−0.54
		Molecular Dynamics							
Final Model	*ABCG2* ^b^	88.602	2.36	55^th^	0.439	−3.264	−1788.05	1-2-2	−1.03
	MODEL1	93.729	1.78	86^th^	0.414	−3.855	−1854.90	1-2-2	−0.65
	MODEL2	95.000	1.64	91^st^	0.413	−3.872	−1878.03	1-2-2	−0.63
	MODEL3	94.992	1.71	89^th^	0.417	−3.831	−1878.03	1-2-2	−0.65

^a^recently published human *ABCG2* cryo-EM structure at 3.8 Å resolution; ^b^previously published *ABCG2* homology model, obtained from http://abcg.hegelab.org (ABCG2_V2.pdb and ABCG2_V2_prteq.pdb).

**Figure 2:**
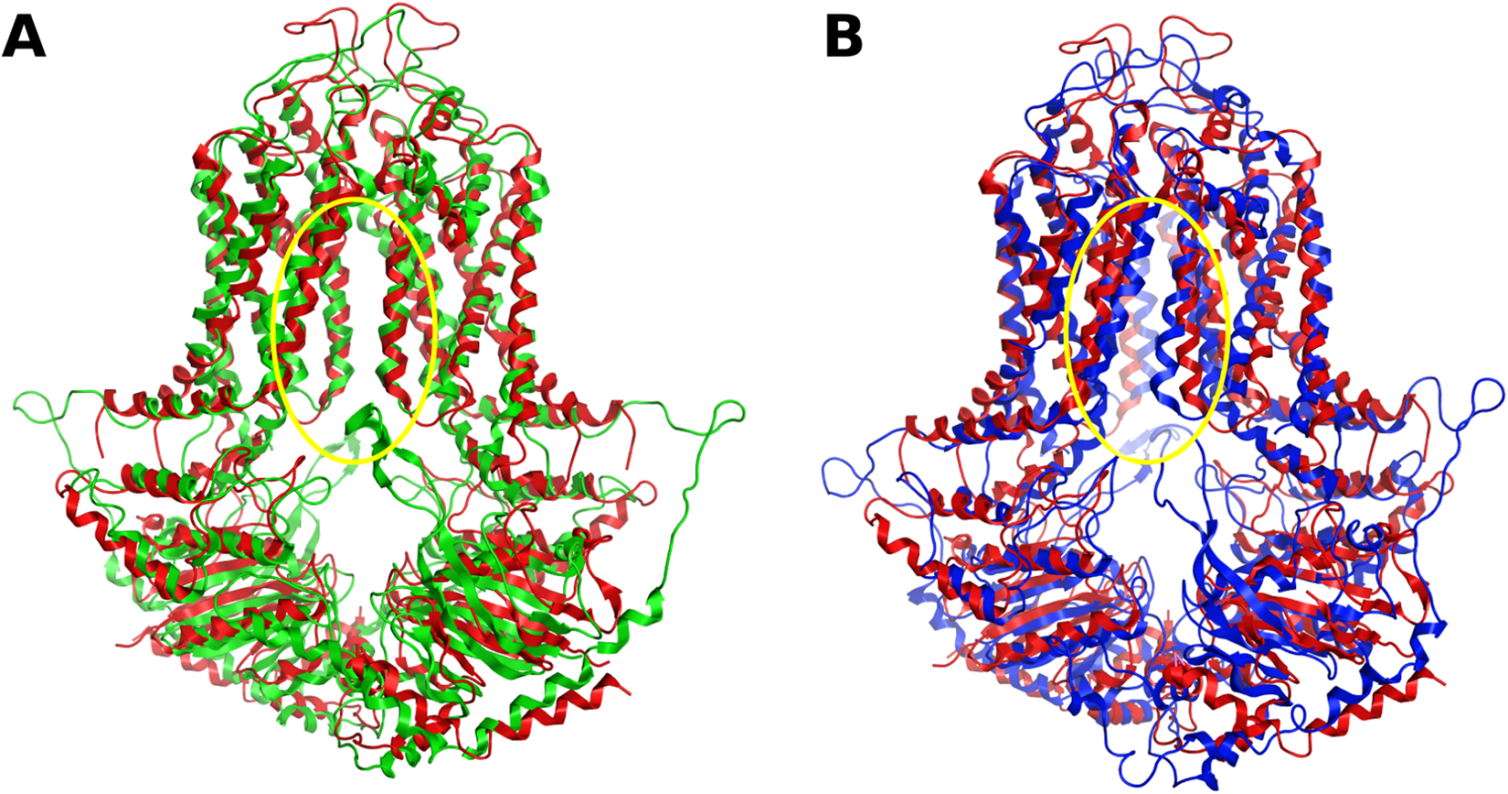
Superimposition of the human *ABCG2* crystallographic structure (PDB ID: 5NJ3) with the homology model (**A**) and the 200 ns MD structure (**B**). The yellow circle annotates the location of the asymmetry due to the backbone shift of TM4 and TM5a.

By the superposition of the structural data of the human *ABCG2* transporter with our homology model, obtained by duplication of the *ABCG5* half transporter (Fig. 2A), all major structural features are found to overlap (monomer RMSD, 2.81 Å; homodimer RMSD, 4.30 Å), with the larger deviations being found at the extracellular coils, the *de novo* modeled intracellular C2 helices and at the top of the TM5a, before the 5b/5c helical bundle found to be characteristic of the *ABCG* family. Moreover, and while a slight backbone shift of TM1b and TM3 were observed, an inward-facing cavity was found to be present in our homology model, similar to the one identified in the *ABCG2* crystal structure due to the backbone shift of TM helices 2 and 5a, but absent in a previously published homology model^42^ obtained from the whole *ABCG5/G8* heterodimeric transporter^41^.

In opposition to a previous model published recently^42^, a significant improvement was observed when compared with the *ABCG5/G8* crystallographic structure. When compared with the human *ABCG2* crystal structure, our refined model still behaves quite well and, despite the lower scores for the G2_MODEL_ and G2_FULL_ in the SwissModel structure assessment tools, the values are comparable with the ones obtained for the crystallographic structure and are in agreement with the stereochemical quality expected for membrane proteins because QMEAN Z-Score uses solvent accessibility as one of its scoring components, which confers poorer scores for membrane proteins and predicts these as lower quality models^43,44^. Furthermore, for membrane proteins Z-scores above −5 are indicative of a good quality model.

When considering the *ABCG2* structures obtained after the 200 ns MD runs, all three models (models 1, 2 and 3) showed better scores when compared with the previously published one. Hereafter, as both models 2 and 3 were evaluated as the best ranked models, the discussion will be focused on the results obtained for model 3 unless stated otherwise. Herein, and when compared with the h*ABCG2* cryo-EM data, the equilibration of the structure induced a distinct asymmetry between both halves, mainly due to the backbone shift of TM4 and TM5a in one half towards TM1b’ and TM2’ of the opposite monomer. However, it is also evident that the overall architecture for the *ABCG2* transporter is maintained (RMSD of 3.18 and 3.48 Å between each of the monomers with the monomeric h*ABCG2*) and that the modeling of the missing A-loop (Y44-L64), the connecting helices C2a-C2b (CnH, G311-S354) and the highly charged linker between the connecting helices CnH and TM1a (G355-Y369) did not have any impact on the structural stability of either the nucleotide-binding domains or the first transmembrane helix (TM1a and TM1b).

**Figure 3:**
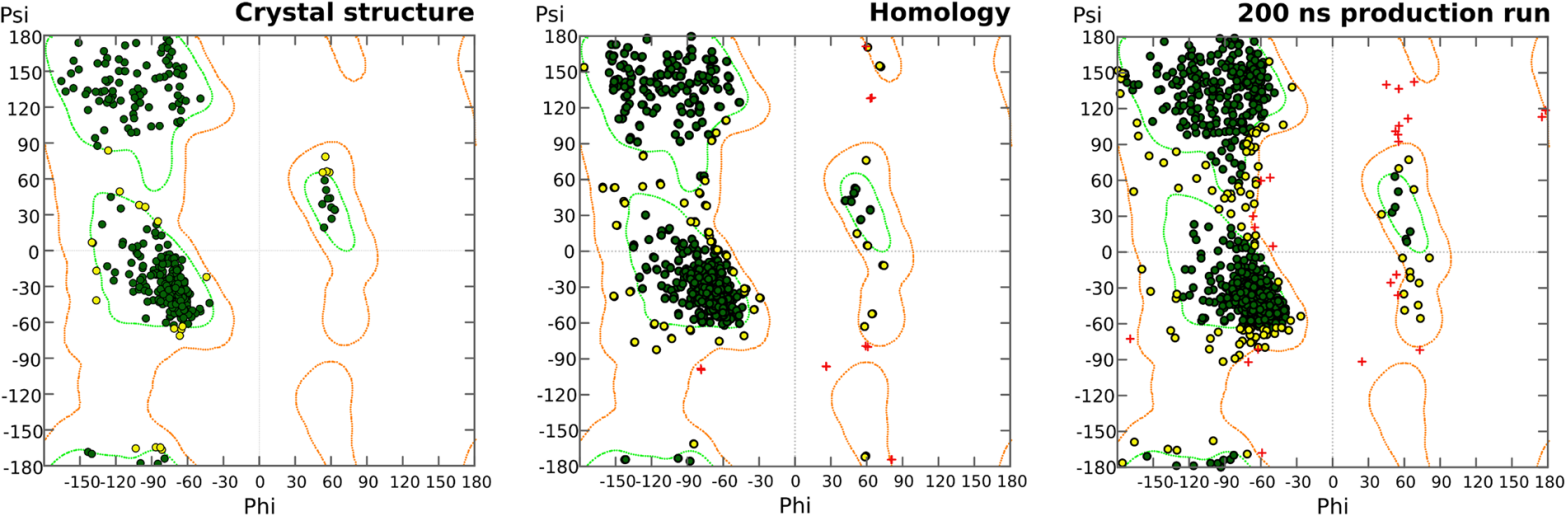
Ramachandran plots of the *ABCG2* crystal structure, homology model and after 200 ns production run. Allowed regions are delimited by an orange curve and favored positions by a green curve.

By comparing the Ramachandran plots between the h*ABCG2* crystal structure, the initial homology model and after the 200 ns MD simulation, and despite the overlapping plot for the initial homodimers, the overall structure of the *ABCG2* is maintained (Fig. 3) with 87.1% of all residues in the most favorable regions, 10.7% on allowed regions and only 2.2% in disfavored regions, in accordance with previous results obtained for the MD simulation of another ABC transporter (P-glycoprotein)^36^. Still, from the 27 outliers, 12 residues can easily shift into the allowed region of the plot (they overlap the contour line that defines the boundaries for such regions) and the remaining 15 are mainly located in coils, except for Phe402 and Ser413 that are located at the second transmembrane helix in chain B.

**Figure 4:**
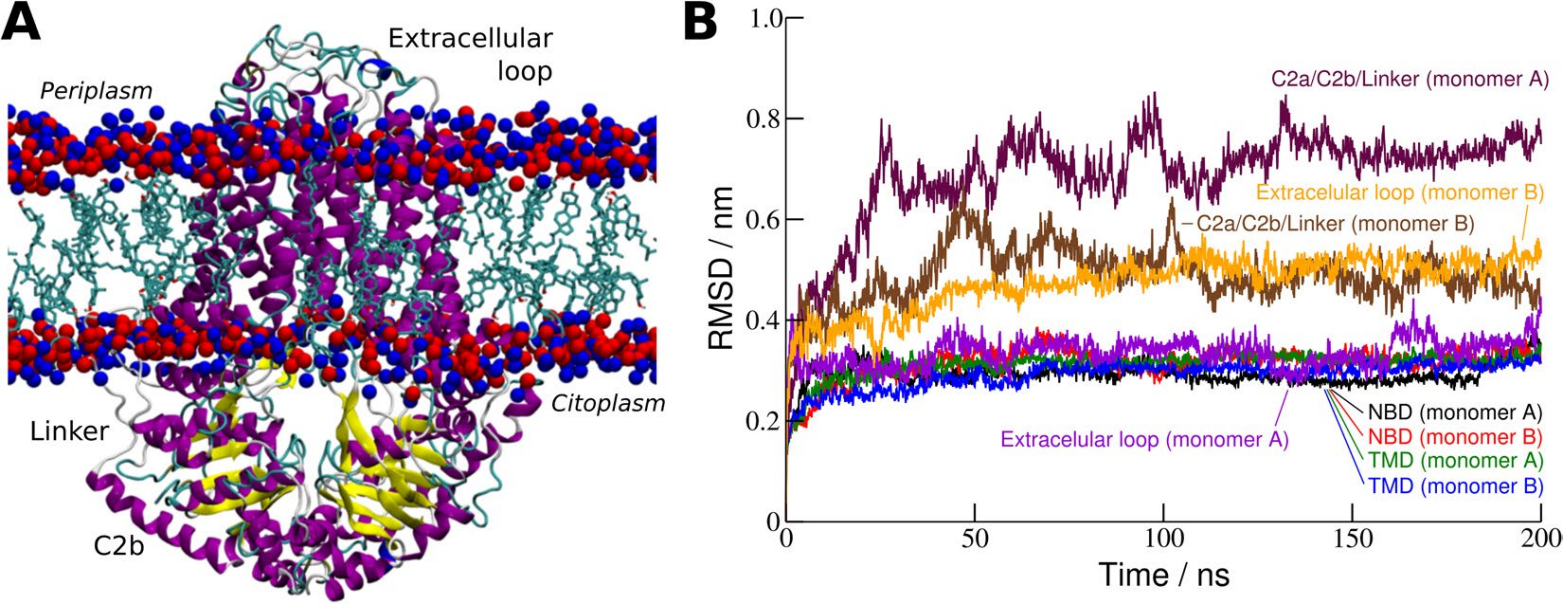
(**A**) Final structure with liquorice representation of the cholesterol residues within the lipid membrane and lipid heads in CPK (red, phosphate; blue, nitrogen); (**B**) RMSD of residue α-carbons grouped by domains in both chains A and B (NBD, nucleotide-binding domain; TMD, transmembrane domain; C2a/C2b/Linker, *de novo* modeled segments.

The root-mean-square deviation (RMSD) is another important parameter for assessing structural deviations from a given reference structure, in this case the initial homology model (Fig. 4). The RMSD value for the whole transporter increased in the first 50 ns, reaching a plateau at ~4 Å that was maintained until the end of the simulation time. When this parameter is plotted for each *ABCG2* domain separately (Fig. 4B), it is clear that in both chains the *de novo* modeled C2a/C2b/Linker domains are the major contributors for the total RMSD. Moreover, in the recently published G5G8 structure, the *ABCG8* subunit have a similar structural organization to *ABCG2* (two α-helices followed by a long linker)^41^. However, even in the most recent *ABCG2* structure such domains were unable to be fully determined by crystallography, which is in agreement with the increased flexibility of these particular regions found in the MD simulations. Figure 4B also clearly shows that the other structural domains (TMD and NBD) remains stable in both subunits, within a maximum RMSD of about ~3 Å.

To further evaluate the overall fold quality of the homology model, we also fitted our 200 ns *ABCG2* structure to i) a previously published low-resolution 3D structure, obtained by electron cryo-microscopy from two-dimensional crystals and in the absence of nucleotides and substrates^49^ and ii) to the recently published three-dimensional cryo-EM density map from which h*ABCG2* was obtained^18^ (data not shown). In the first case, by overlapping our model into the cryo-EM electron density map while changing the contour level to a value closer to the mean density (σ = 0) we were able to obtain a good fitting with the electron density, in better agreement than the previous homology models built using P-gp as a template^50,51^. Moreover, and by using the most recent cryo-EM electron densities from h*ABCG2*, while the transmembrane domains were found to fit within the electron densities corresponding to the membrane-embedded alpha-helical domains deeply buried into the lipidic nanodiscs, the *de novo* modeled segments were found to correspond to the electron density next to both nucleotide-binding domains attributed to helical segments C2a and C2b. Interestingly, and unlike C2a/C2b/Linker motifs, it was observed that the position of the helix 1a remained stable at the membrane interface throughout the whole MD simulation while simultaneously showing the ability to “slide” through the interface, accompanying the positional shifts of helix 1b, which may mean that this particular segment may have an important function in the efflux conformational dynamics of *ABCG2*.

### Disulfide bridges, cholesterol binding and ATP hydrolysis

In this homology model, no disulfide bonds were modeled because i) intermolecular disulfide bond between cysteine residues in position 603 are not required for its transport function^19,21^, ii) intramolecular disulfide bonds may exist but, while not essential for *ABCG2* localization and transport activity^20,23^, are a key factor in determining the fate of *de novo* synthesized proteins^24^ and iii) cysteine-less transporters can still be expressed and targeted to membranes, with internal cysteines being more important than the ones located at each ECL^52^.

We measured the Cα-Cα distance between cysteines 592 and 608 (intramolecular) or between cysteines 603 in both chains (intermolecular) to assess if the distances are compatible with the existence of disulfide bonds. In addition, due to the absence of physical bonds between both *ABCG2* monomers, we also assessed their relative free energy of binding between subunits with *g_mmpbsa* added with the implicit membrane correction. While considering intramolecular bridges (C592-C608), Cα-Cα distances ranged from 0.60 ± 0.07 nm (model 1) up to 1.26 ± 0.07 nm (model 3), and are therefore compatible with the physiological existence of disulfide bonds between both residues. For the intermolecular bridge, Cα-Cα distances were found to be 0.95 ± 0.10 nm (model 1), 1.36 ± 0.15 nm (model 2) and 1.05 ± 0.23 nm (model 3), again compatible with intermolecular disulfide bond formation when in an oxidative environment and also in agreement with previous cited experimental data.

Similarly, for cysteine residues at position 284, also thought to be involved in intermolecular bridges responsible for a dimeric protein with slow mobility^52^, mean Cα-Cα distances ranged from 1.53 ± 0.17 nm (model 3) up to 1.76 ± 0.07 and 1.83 ± 0.08 nm in models 1 and 2 respectively, in good agreement with the above experimental results. Moreover, the location of the remaining cysteines do not allow the formation of either intra or intermolecular disulfide bonds, but nonetheless any mutations in these residues are expected to affect NBD:TMD communication (C438, located close to the coupling helices in TM helix 2) or signal propagation (C374, at the end of the ‘linker’) without compromising substrate specificity^52^. Finally, free energies of binding between both *ABCG2* monomers for the last 50 ns of the final MD simulation were calculated to be −1124 ± 63 kcal.mol^−1^, lower than the value calculated for the G5G8 heterodimer (−902 ± 59 kcal.mol^−1^, unpublished data).

**Figure 5:**
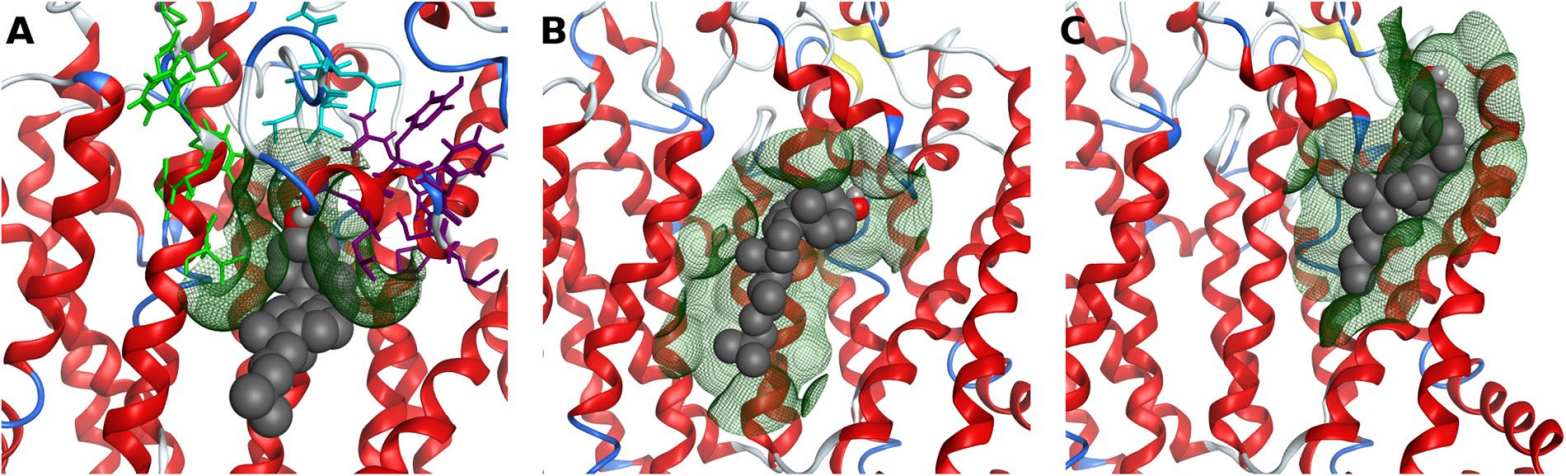
Cholesterol binding to *ABCG2*. (**A**) Cholesterol close to LxxL (cyan) and CRAC domains Y413 (green) and Y570 (purple); (**B**) cholesterol bound at the dimer interface; (**C**) cholesterol bound to CRAC Y570 in the second *ABCG2* subunit. Protein surface is represented by a dark green mesh.

Several motifs in *ABCG2* were also described to  be important for its cholesterol sensitivity^30^, ATP binding and hydrolysis^14,25,26^ or substrate binding^53^. Regarding cholesterol, Gál *et al*.^30^ showed that while mutations in the LxxL motif (aa 555–558) resulted in an apparent cholesterol insensitivity, only one of the five identified CRAC domains (Y413) was sensitive to the presence of cholesterol. Interestingly, in *ABCG2* both domains (LVNL and VIGAIYFGLK) are located close to each other (Fig. 5A) and next to an additional cholesterol insensitive CRAC domain (Y570, LSWLQYFSIPR)^30^. More relevant, a cholesterol molecule was found to be located in this particular region, close to all three motifs and making a hydrogen bond with Q569. Herein, the relative free energy of binding for cholesterol was calculated to be −57 ± 5 kcal.mol^−1^, against only −40 ± 6 kcal.mol^−1^ when located closer to the dimer interface (Fig. 5B). In addition, another cholesterol molecule placed in the symmetrical location (near the Y570 CRAC motif in the other *ABCG2* monomer, Fig. 5C) also showed a similar relative free energy of binding of −56 ± 6 kcal.mol^−1^. Therefore, our results clearly show that such regions are involved in cholesterol binding and are also in agreement with the electron density data published by Lee *et al*.^41^ for the G5G8 transporter in which some features in the electron density map suggest that cholesterol is bound at this location.

**Figure 6:**
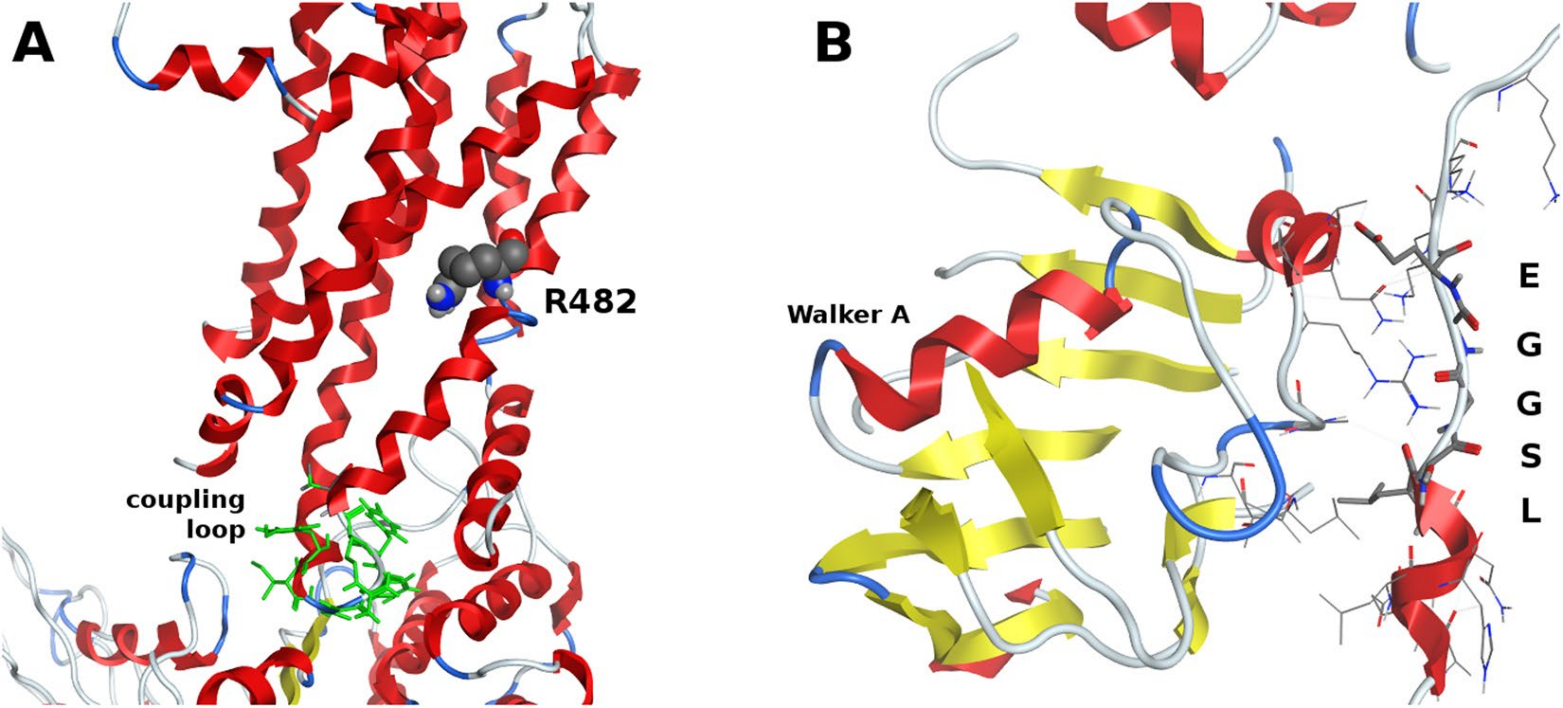
(**A**) Location of the arginine 482 and coupling loop (green) in the *ABCG2* structure; (**B**) Location of the C2-sequence (LSGGE) and residue interactions with NBD residues.

When considering ATP binding and hydrolysis, mutations at position R482^25,26^ or in a second signature motif (C2-sequence, LSGGE)^14^ were also proved to disturb ATP hydrolysis. However, while R482 is located in transmembrane helix 3, and thus connected to Walker A through the NBD coupling loop (TMH2-loop-TMH3, Fig. 6A), the C2-sequence is located in the beginning of the linker, anchoring the preceding helix to the NBD and also in contact with the internal helical domain containing the Walker A motif (Fig. 6B). Thus, structural modifications in these two regions will have a direct impact on the signal propagation between nucleotide-binding and transmembrane domains, with little impact on drug binding.

Finally, considering drug binding and transport, mutations in proline and methionine residues at positions 485 (P485A) and 549 (M549A) respectively were found to be related with differences in the ability of *ABCG2* to transport several substrates^53^. While P485 is located in the same helix as R482, M549 is located in the vicinity of the LxxL and CRAC motifs, described above as part of a binding site for cholesterol. Therefore, such location can also be a possible drug-binding site for *ABCG2* substrates (more details, in the *Docking* section below). Moreover, the A540F mutation in the *ABCG5*, which is closely related with the M549A in the *ABCG2* structure, also impaired cholesterol transport without affecting G5G8 heterodimer expression^41^.

### Protein global motions

**Figure 7:**
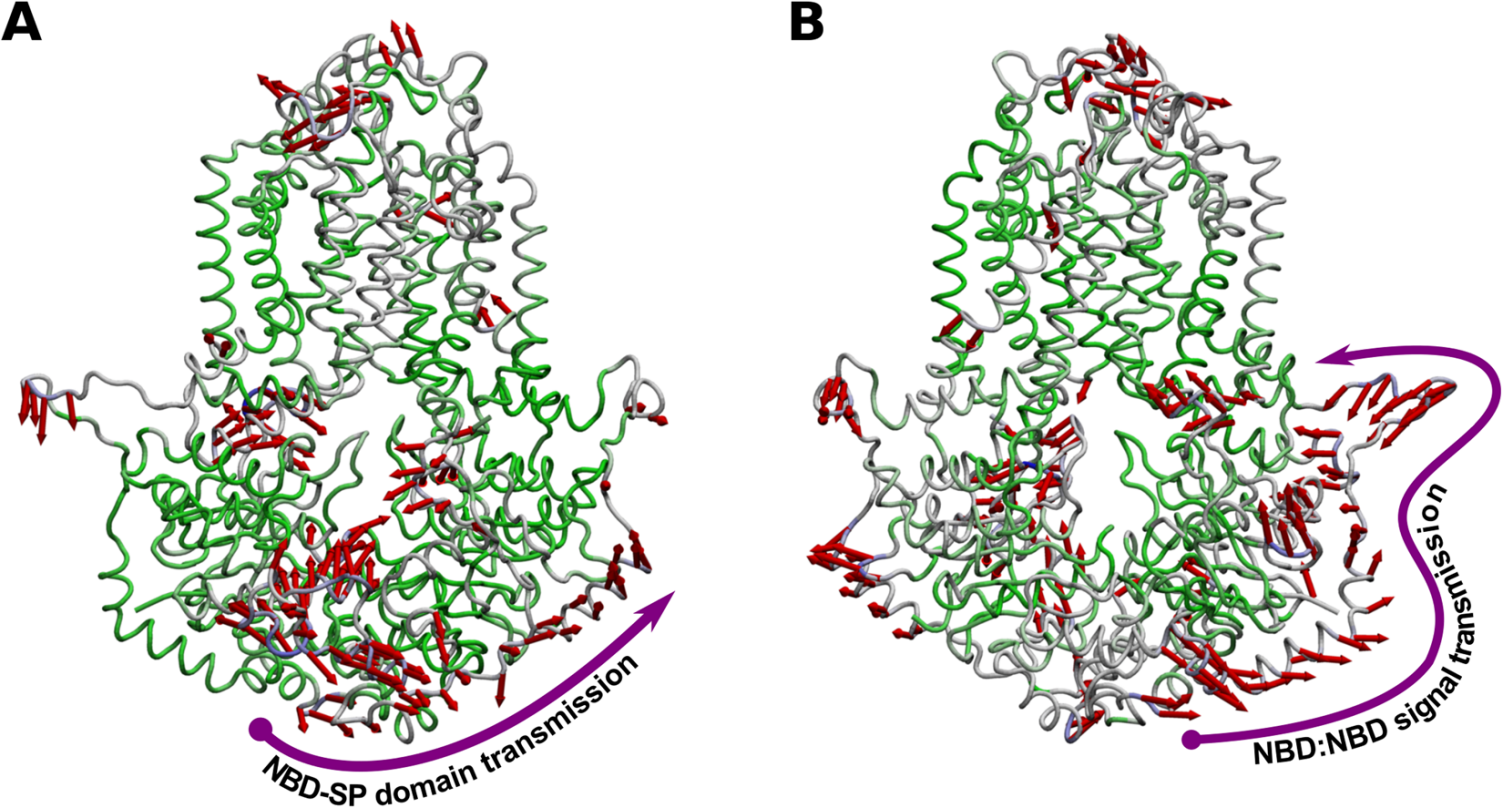
Normal motion patterns displayed by *apo ABCG2*. (**A**) Mode 1 in *ABCG2* principal component analysis; (**B**) Mode 2 in *ABCG2* principal component analysis. blue- and green-colored regions correspond to high and low mobility domains, respectively.

Being *ABCG2* a homodimeric transporter, we were particularly interested on how conformational changes could propagate through the structure. In a previous study of another ABC transporter (P-gp), it was found that the ‘linker’ domains acts as a ‘damper’ in order to reduce NBD fluctuations and that, in the presence of substrates, a clear change to efflux-like motions could be observed^36^. In *ABCG2*, however, and due to the absence of a linker connecting both halves, signal transmission between monomers occurs through the external helical domains (residues 305–351), acting as a ‘spring’ in transmitting the motions from one NBD to the other (Fig. 7).

In the most predominant mode 1 (Fig. 7A) it is possible to verify that motions in one NBD are propagated to the opposite NBD by a direct interaction with its external helices (although with a minimal impact on its core), while mode 2 shows that motions originated at the NBD:NBD interface are also propagated through these helical segments, reaching the membrane-anchored portion of the linker (Fig. 7B).

Thus, if specifically involved in signal propagation, it is expected that these domains (hereby named signal-propagating domains, or SP domains) should be extremely mobile, quickly shifting between conformations in order to better propagate the signal between monomers. Interestingly, similar domains are also incomplete in the crystallographic *ABCG8* structure, which corroborates these assumptions. It was previously proposed that the A-loop, also missing in both *ABCG5* and *ABCG8* structures, could act as a ‘filter’ at the entry of the substrate cavity or by contributing as the first step of allosteric communication between drug binding and ATP binding^42^. However our results show that, in both monomers, the A-loop is anchored at the membrane interface through residues 48–55 (LKSGFLPC) and, therefore, they seem unable to participate in allosteric interactions with substrates.

**Figure 8:**
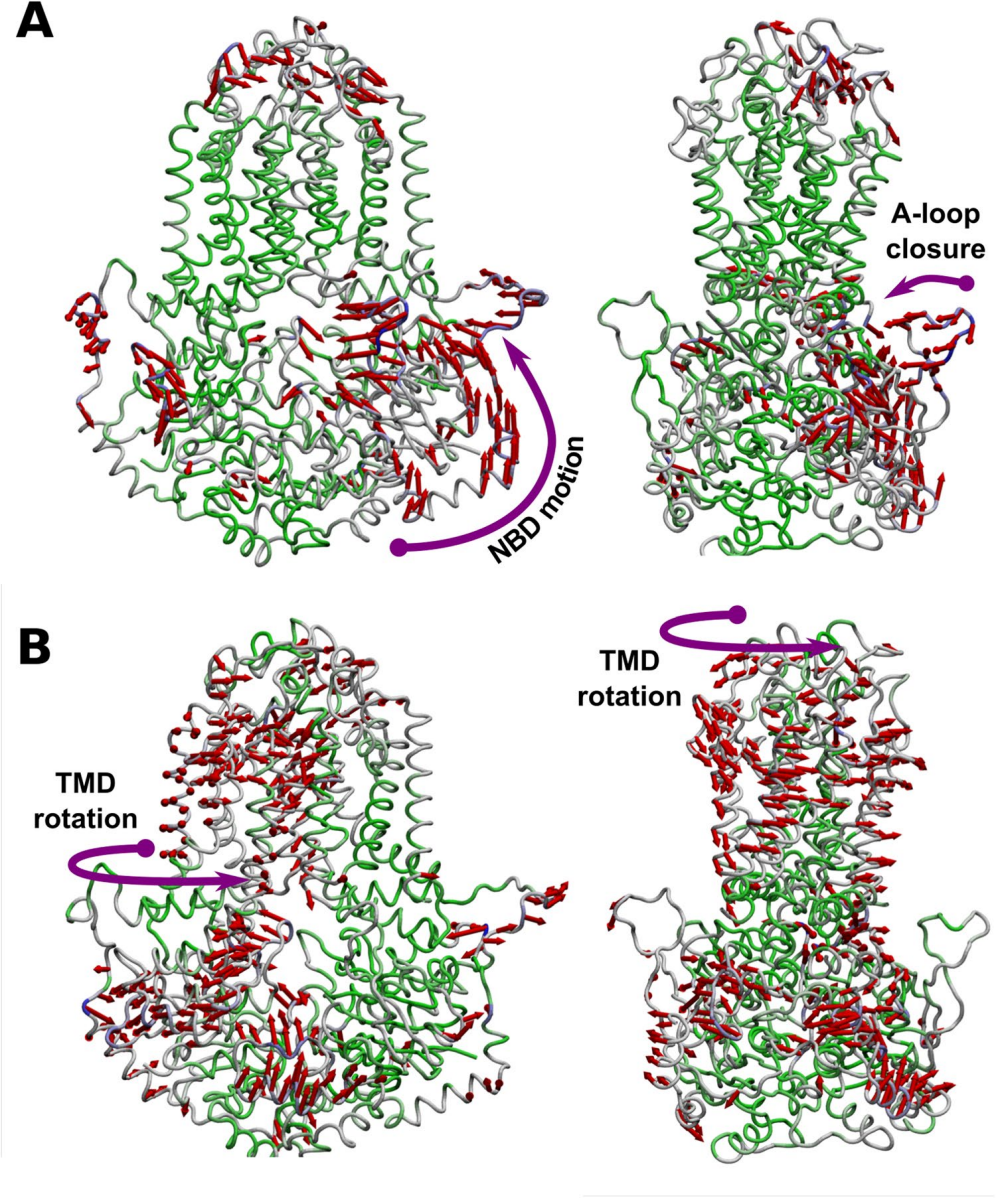
Normal motion patterns displayed by *holo ABCG2* in the presence of ATP. (**A**) Mode 1 in *ABCG2* principal component analysis; (**B**) Active mode 3 in *ABCG2* principal component analysis. blue- and green-colored regions correspond to high and low mobility domains, respectively.

However, in the presence of ATP (Fig. 8), mode 1 of the principal component analysis distinctly shows an upward motion of one NBD in which the A-loop moves closer to the opposite NBD, i.e. closing the access to the ATP-binding site and promoting interactions between the Walker A and Signature motifs, together with a coordinate movement of the SP domains that propagate the signal forward to the transmembrane domains (Fig. 8A). Moreover, active modes 2 and 3 additionally shows distinct efflux-like motions, characterized by NBD_A_:NBD_B_ distance fluctuations (active mode 2) and rotation of the TM helical bundle (active mode 3, Fig. 8B), similar to that observed for P-gp^36^ or G5G8 heterodimer (unpublished data). Therefore, A-loop and SP domains seem to be crucial in the ATP-dependent efflux cycle: while the A-loop act as a “cover” for the ATP-binding site, favoring ATP binding and enhancing the contact between Walker A and Signature motifs upon NBD dimerization, SP domains are involved in signal transmission, either between the nucleotide-binding domains (in its *apo* form) or after ATP binding to the NBD (*holo* form).

### Molecular docking

**Figure 9:**
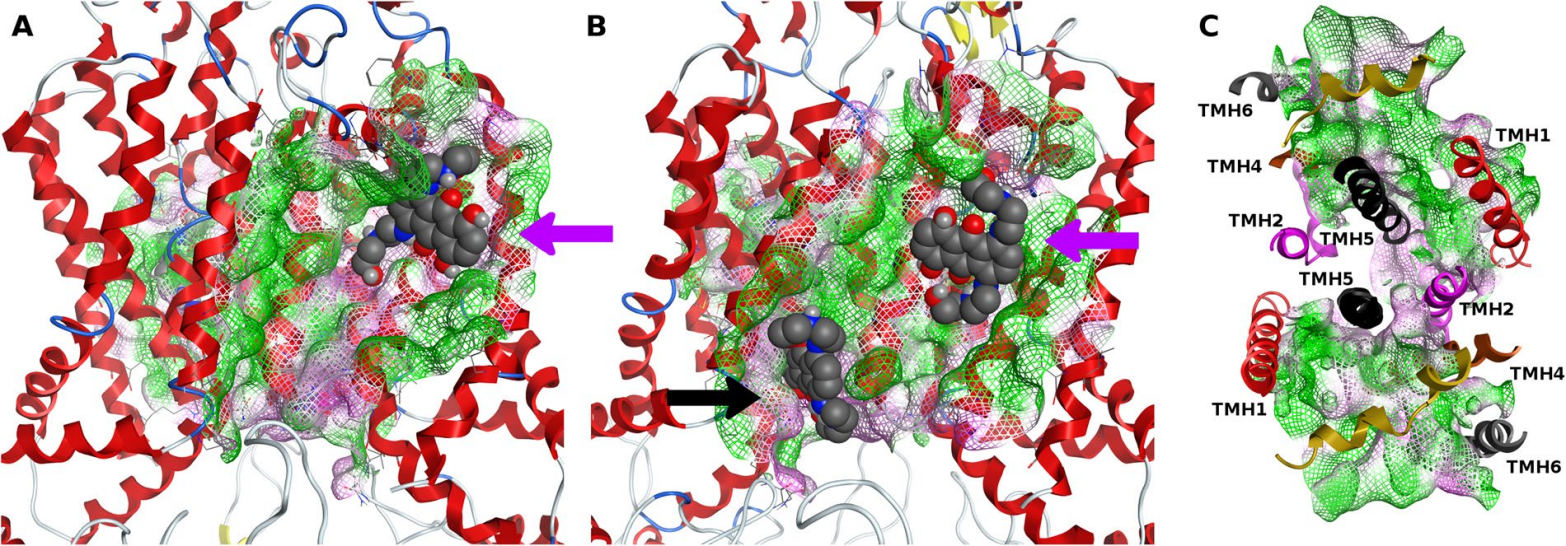
Graphical representation of the Molecular Surface (MS, green, hydrophobic and pink, polar) for the identified drug-binding sites in the first (**A**) and second (**B**) *ABCG2* monomers. (**C**) Transmembrane helices organization around the identified drug-binding sites.

Kinetic and equilibrium data by Clark *et al*.^54^, suggest the presence of at least two symmetric drug-binding sites on *ABCG2*, one in each monomer, although displaying allosteric communication between them. Due to the large substrate overlapping with P-gp, it is also expected that *ABCG2* extrudes its substrates from the lipid bilayer after membrane partitioning from the cytoplasm^55^. Therefore, in our study the whole TM domains (embedded within the membrane) were used to define the docking box and a known substrate, mitoxantrone (MX), was used to sample possible drug-binding sites within the *ABCG2* transporter (Fig. 9). Three drug-binding sites could be identified from the best docking poses for mitoxantrone: although the last one (Fig. 9B, black arrow, −5.7 kcal.mol^−1^) is in agreement with the electron density reported for cholesterol in the recently published human *ABCG2* cryo-EM structure^18^, the other two sites are herein described for the first time (Fig. 8A,B, purple arrows, −6.3 and −5.8 kcal.mol^−1^ respectively). The latter two, located in each monomer, comprise a surface ‘groove’ immediately below a short helical segment bearing a cholesterol-insensitive CRAC domain (residues 565–575) that is part of the long extracellular loop connecting helices 5 and 6 (named “plug” in the h*ABCG2* structure).

**Figure 10:**
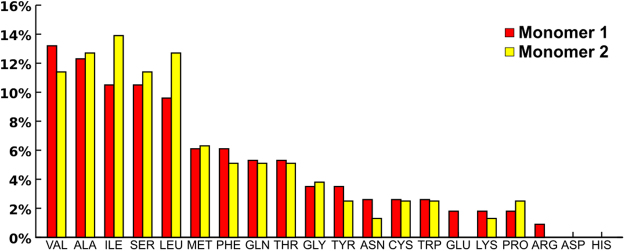
Residues distribution percentage for each binding cleft.

Quite interestingly, these particular sites are also deeply buried within the membrane^55^, show a distinct symmetry between monomers^54^ and share common residues with the above described cholesterol-binding site^56^, suggesting that the drug-binding sites in *ABCG2* may encompass the large majority of the membrane-facing surface of the transmembrane helical segments to allow binding of larger molecules than cholesterol. Indeed, a close inspection of the top-ranked binding poses for other *ABCG2* substrates as pheophorbide A (−7.7 kcal.mol^−1^), flavopiridol (−7.7 kcal.mol^−1^) or 9-aminocamptothecin (−7.8 kcal.mol^−1^) show that these sites are located in a larger ‘surface cleft’ with approximately 1900 A^3^ (as estimated by EPOS^BP^) spawning from the CRAC-insensitive helical segment above described to the center of the dimer interface (reported for cholesterol by Taylor *et al*.^18^) and also in close agreement with the sterol-binding sites proposed for *ABCG5/G8* ^41^. Each drug-binding site is flanked by TM helices 1-2 of the opposite monomer, by TM helices 4–6 and, on top, by a small helical domain that is part of the large extracellular loop between TM helices 5 and 6 (Fig. 9C). Interestingly, it was also verified that, in our model, the second transmembrane helix of one of the monomers is involved in both drug-binding sites, suggesting some degree of allosteric communication between both sites as suggested by Clark *et al*.^54^ The identification of these large ‘surface clefts’ within the *ABCG2* structure as the hypothetical drug-binding sites led us to thoroughly characterize its lining residues, mean relative polarities and volumes through EPOS^BP^ software. Unlike P-gp, where M-site and R/H sites are characterized by a greater number of aromatic and polar residues respectively^32^, *ABCG2* binding clefts have instead a larger number of hydrophobic residues as valine, alanine, isoleucine and leucine (Fig. 10). In each monomer, hydrophobic residues account for 60 ± 3% of all residues against 29 ± 2% of polar and only 11 ± 1% of aromatic residues, but while hydrophobic residues are scattered through the whole cleft, polar residues are more abundant in helices close to the dimer interface (most particularly in TM helices 1 and 2) whereas aromatic residues are found at the top of the pocket, in the short α-helix that precedes the ECL between TM helices 5 and 6.

**Figure 11:**
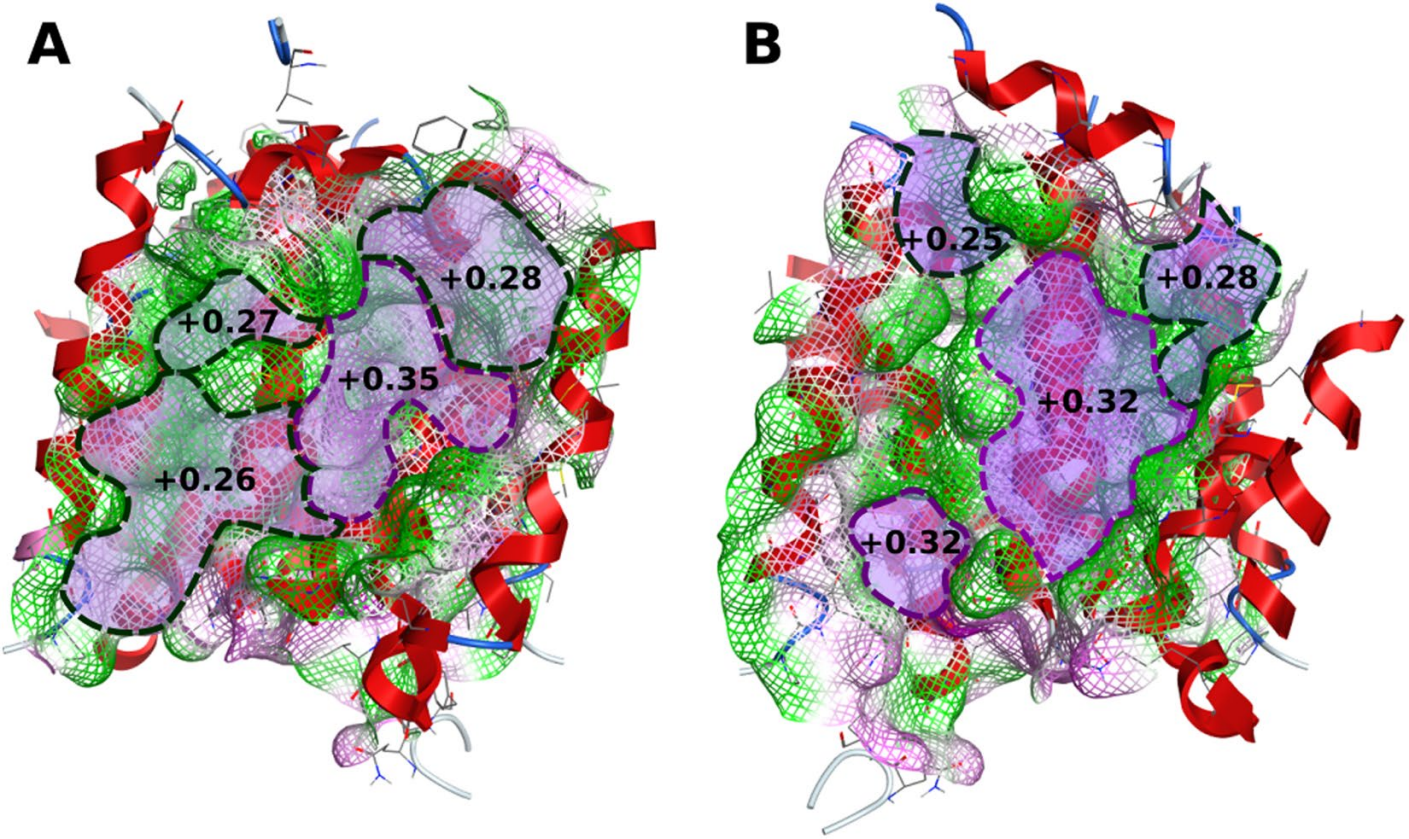
Mean polarities of both binding clefts in monomer A (**A**) and B (**B**).

Moreover, both binding clefts have similar mean polarities ( +0.32 and +0.29) when compared with those found for P-gp ( +0.32 and +0.33 for R and H sites respectively). Yet, some differences could still be found within the drug-binding sites due to the asymmetrical residue distribution (Fig. 11). First, while the center of the cleft is more polar ( +0.35 or +0.32 in each monomer), the uppermost region of the site is more lipophilic (ranging from +0.25 to +0.28). Second, the spatial position of the first transmembrane helix affects the polarity of the drug-binding site near the dimer interface. As in monomer A TM helix 1 of the opposite monomer is closer to TM helix 5, the dimer interface is less hydrophilic, with a mean polarity value similar to the one observed at the top of the drug-binding site ( +0.26, Fig. 11A).

**Figure 12:**
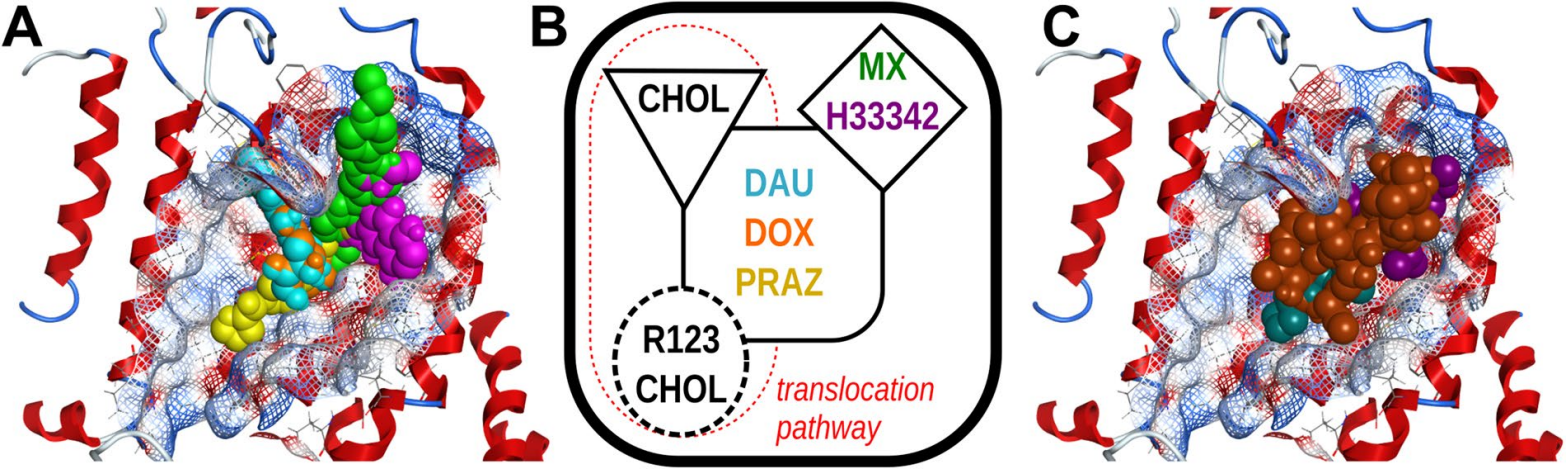
(**A**) Top-ranked docking poses for substrates: mitoxantrone (MX, pink), Hoechst 33342 (H3342, green), daunorubicin (DAU, cyan), doxorubicin (DOX, orange) and prazosin (PRAZ, yellow) at the drug-binding site in monomer A, **(B**) Proposed schematic representation of *ABCG2* drug-binding sites, based on a previous model by Clark *et al*. and (**C**) Top-ranked docking poses for modulators: cyclosporine (CYC, brown), fumitremorgin C (FTC, purple) and KO143 (dark green). The mesh represents the drug-binding pocket surface and is colored by the electrostatic potential (blue, electron donor; gray, neutral and red, electron acceptor).

However, a different conformation in monomer B induced by the lateral shift of TM helix 1b exposes the more hydrophilic core of TM helix 5 and creates an additional site with a mean polarity similar to the DBS center ( +0.32) and providing an additional binding site where cholesterol could be found^18^. Thus, as in P-gp^32^, these distinct polarities within the binding cleft may be one possible explanation for the results obtained by Clark *et al*.^54^ in which, although using the R482G variant, several substrates appear to bind to distinct locations (Fig. 12A). By combining this information with the results for cholesterol (obtained from both the MD simulations and molecular docking) and with Rhodamine-123 (R123) docking poses in monomer B (at Site 2, −6.7 kcal.mol^−1^ against −7.4 kcal.mol^−1^ for the top-ranked binding pose), a new schematic representation of the *ABCG2* drug-binding sites, based on the one previously proposed by Clark *et al*., could be generated (Fig. 12B). Hence, while mitoxantrone (MX, −6.3 kcal.mol^−1^) and Hoechst33342 (H33342, −8.9 kcal.mol^−1^) bind near the top of the drug-binding site, daunorubicin (DAU, −7.9 kcal.mol^−1^), doxorubicin (DOX, −7.8 kcal.mol^−1^) and prazosin (PRAZ, −7.4 kcal.mol^−1^) are found to bind closer to the center of the ‘groove’.

Concerning R123, although a specific site is proposed to exist close to the dimer interface (as for mitoxantrone) in the wild-type protein, our data together with the recently reported cholesterol localization in the human *ABCG2* crystal structure suggests that this location would alternatively be part of the translocation pathway (Fig. 12B, red dashed line). Therefore, one possible explanation for the increased R123 efflux described for the R482G variant^16,42^ may be that the Arg → Gly mutation at position 482 induces a permanent shift on the position of TMH 3 towards TMH 4 while moving away from TMH 1, which would allow the formation of the above identified cavity between TMH1 and TMH5 (as seen in one of the monomers, Fig. 11B) where R123 was found in our docking procedure. Finally, a distinct site for cholesterol could also be identified in the vicinity of the active Y413 CRAC motif (Figs. 5A-B, docking, −7.9 kcal.mol^−1^), in agreement with experimental studies in which cholesterol influence the binding process of *ABCG2* substrates^27^ as an allosteric co-activator or through co-transport with substrates^28^ and in agreement with the results found for the heterodimeric sterol transporter *ABCG5*/*G8* ^41^.

### Membrane data

As cholesterol is a crucial component of lipid bilayers and is important for the activity of the *ABCG2* transporter, we measured important parameters as the protein’s angle of insertion (tilt), area per lipid (*A*_L_), thickness (*D*_HH_) and cholesterol distribution around the *ABCG2* transporter to assess the influence of the transporter on the surrounding lipid environment. Regarding the membrane insertion angle (tilt), in our systems *ABCG2* is found to have a 3 ± 1° tilt, which is in agreement with the low tilt angle predicted by OPM database (6°). As P-gp, another member of the ABC transporter family that is known to reshape the surrounding lipid environment^57,58^, *ABCG2* was found to have a strong influence on the membrane. When comparing the area per lipid (*A*_L_), and taking into account the values for pure DMPC (0.602 nm^2^) and with 20 molar-% cholesterol (0.531 nm^2^; partial areas of 0.563 and 0.396 nm^2^ for DMPC and cholesterol respectively), it was found that the presence of *ABCG2* increases the *A*_L_ up to values similar to pure DMPC membranes (0.591 nm^2^), corresponding to a 10% increase on the membrane’s mean areas per lipid (partial areas of 0.661 and 0.316 nm^2^ for DMPC and cholesterol, respectively). Similarly, although membrane thickness also decreases (3.62 nm) when compared with 20 molar-% CHOL:DMPC membranes (3.98 nm), it does not reach the values for pure DMPC (3.27 nm). We revisited our previous studies on P-glycoprotein-membrane systems^36^ and observed that while *A*_L_ values shifts from 0.636 nm^2^ (pure POPC) to 0.593 nm^2^ (20% CHOL:POPC, −7%), in P-gp/bilayer systems comprising cholesterol (system built from the refined model but not included in ref^15^) the *A*_L_ is 0.612 nm^2^ (3% increase). Regarding thickness, in the presence of P-gp the calculated thickness of a 20% CHOL:POPC membrane was 4.01 nm, against 3.79 nm for 20% CHOL:POPC membrane ( +5%) and 3.72 ( +7%) for pure POPC. Therefore, these data show a stronger effect by *ABCG2* in the surrounding lipid environment when compared to P-gp.

As it is known that cholesterol reduces membrane fluidity by increasing the orientational order of the hydrophobic chains, reducing its area per lipid and by increasing its thickness^59,60^, this led us to hypothesize that the modulation of cholesterol content within the membrane^27,61^ affects the *ABCG2* dimer cohesion due to a decrease on the membrane’s lateral tension. This hypothesis also provides a suitable explanation for the irreversible dissociation of *ABCG2* dimers by detergents, as previously shown by Telbisz *et al*.^28^. This also suggests that *ABCG2* has a higher dependency on the biophysical properties of the membrane.

**Figure 13:**
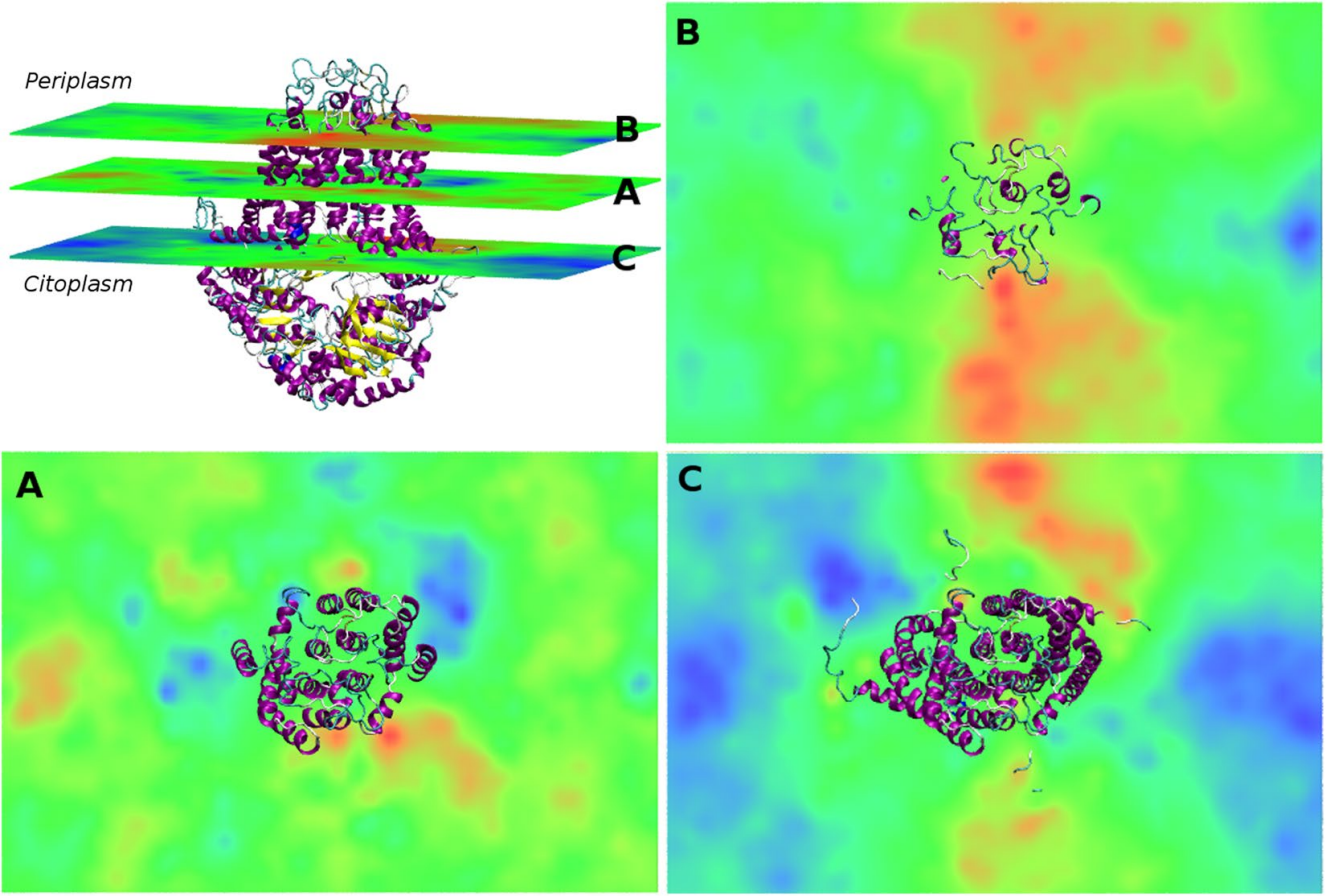
Volumetric thickness maps for the *ABCG2*/membrane systems. (**A**) average thickness map (**B**) deformation map for the upper leaflet and (**C**) deformation map for the lower leaflet. *ABCG2* is depicted in cartoon representation^63^. Negative and positive deformations are depicted in a blue-red scale for membrane contraction and expansion, respectively.

In addition to the above results, we also generated volumetric maps interpolating the *z* coordinates of selected atoms (phosphate atoms) into a regular orthogonal grid in the *xy* plane and superimposing them with the *ABCG2* transporter^62^ (Fig. 13). Quite interestingly, from the average thickness map (calculated from the whole membrane; Fig. 13A), it is possible to identify regions of thicker membrane patches next to the cholesterol recognition/interaction amino acid consensus, corresponding to an increase in the number of cholesterol molecules and closer to the previously identified drug-binding sites, along with thinner patches on the vicinity of TM helix 6. However, by observing the deformation maps for the periplasmic (Fig. 13B) and cytoplasmic (Fig. 13C) leaflets it could be identified two symmetrical membrane ‘patches’ with increased thickness, both having a funnel-like shape, from which molecules can access, for each monomer, to the surface clefts where the drug-binding sites are located.

## Conclusion

Since the identification of the ABC transporters role in multidrug resistance (MDR) that efflux pumps as P-glycoprotein, MRP1, BCRP (also known as *ABCG2*) and, more recently *ABCB5*, are being thoroughly studied in order to better understand their mechanism of action, aiming the development of potent and selective MDR modulators to avoid chemotherapy failures.

Unlike P-glycoprotein^64^, the lack of suitable crystallographic structures for *ABCC1* and *ABCG2* has severely compromised the knowledge on these two types of ABC transporters. The recent publication of a crystallographic structure of the ABCG family, the ABCG5/G8 heterodimeric transporter, revealed for the first time a new transmembrane arrangement that is characteristic of the ABCG transporters. Although incomplete and despite the low structural identity with *ABCG2*, it could still be a proper template for modeling *ABCG2*, thus allowing new insights on the structural dynamics for this class of transporters. Herein, we report on a new *ABCG2* homology model from a *ABCG5* homodimer (obtained by duplication of the C chain of the G5G8 crystallographic structure)^41^. This was performed to avoid possible asymmetries due to the utilization of a heterodimeric transporter as a homology template. Indeed, we also provided evidences that the herein newly obtained model is comparable to the recently published human *ABCG2* cryo-EM structure and that it performed better than a previously published one^42^, when evaluated using available structure assessment and validation computational tools. It is also important to refer that i) our model and all the above results were obtained prior to the release of the cryo-EM h*ABCG2* structure and ii) due to the release of the cryo-EM structure, we felt the need to rewrite some parts of this paper in order to validate our *in silico* approach for the development of a *ABCG2* homology model. Both our approach and our model are now supported through a thorough comparison with the structural information that could be retrieved from the novel cryo-EM structure and, more important, our paper provides new and valuable information that can be further used to better understand i) the structural dynamics of the ABCG transporters family, ii) possible drug-binding sites within the ABCG2 structure and iii) the intimate relationships between the surrounding lipid bilayer and the transporter.

In our homology model no disulfide bonds were modeled, because neither intra- or intermolecular disulfide bonds are required for the *ABCG2* transport activity^19–24^. Even so, Cα-Cα distances between cysteine residues involved in intra- (592 and 608) or intermolecular (603) bridges were found to be stable, and at distances compatible with the physiological formation of disulfide bonds in *ABCG2* homodimeric complexes. Thus, our results combined with previous published experimental data suggests that inter- and intramolecular disulfide bridges involving C592, C603 and C608 are more important for specific mechanisms as membrane targeting^20^ or monoclonal antibody binding^65^, rather than directly affecting the expression, function or activity of the transporter.

Signal propagation through the homodimeric structure was also assessed through a principal component analysis, in order to better understand how conformational changes can lead to substrate efflux. Our simulations demonstrated for the first time that signal transmission occurs through the herein *de novo* modeled external helices (absent in the crystallographic structure) that connect the nucleotide-binding domain with the first transmembrane helix. Accordingly, we suggest that drug binding and/or ATP binding is able to induce spring-like movements that propagate conformational changes through these specific domains i) into the opposite NBD or ii) into the TM domain of the same monomer. Thus, the development of small molecules targeting these specific motifs may become a promising alternative to develop novel and specific *ABCG2* efflux modulators able to tackle MDR in cancer by impairing drug-induced signal transmission, similar to what was recently suggested for P-gp^66^.

Molecular docking revealed two symmetric drug-binding clefts, one in each monomer, in agreement with a previous study by Clark *et al*.^54^. These two membrane-exposed clefts are flanked by TMH1-2 of the opposite monomer and by TM helices 4–6 and occupy almost all the buried TM surface, with a mean volume of ~1900Å^3^ and regions of distinct polarities, which may explain why substrates seem to bind to distinct locations within this region. Thus, a new schematic representation of the *ABCG2* drug-binding sites is herein proposed, based on a previous scheme by Clark and co-workers^54^, where a distinct region for cholesterol binding (based on MD and docking results) and a translocation pathway can be proposed. Interestingly, as our results suggest the existence of distinct drug-binding sites for substrates, modulators and cholesterol, it theoretically would be possible to develop specific *ABCG2* efflux modulators that i) could specifically compete with cholesterol (thus reducing the activity of the pump), ii) binds in a different location with high affinity while simultaneously being able to block the substrate-binding site and iii) could specifically bind to the whole “surface cleft” in order to impair conformational changes by increasing the structural cohesion of the transmembrane helical domains (as observed for tariquidar in P-gp)^32,67,68^.

Finally, like other ABC transporters that also reshape the surrounding lipid environment^2^, the biophysical properties of the membrane patch surrounding *ABCG2* were also assessed. Important changes in area per lipid, thickness and membrane deformation profiles were observed that may explain the strong dependency of *ABCG2* for cholesterol-enriched membranes. Interestingly, a thicker “funnel-like” shape region within the membrane was identified from which molecules can access, for each monomer, to the surface clefts where the drug-binding sites are located. As the presence of detergents are often associated to function impairment in ABC transporters^28,69,70^, our results also corroborate that reducing its cholesterol content and increasing membrane fluidity may have a deleterious effect not only in protein function but also regarding drug access to *ABCG2* from the lipid bilayer.

## Material and Methods

### Initial Structures and Software

The *ABCG5/G8* heterodimeric crystal structure (PDB ID: 5DO7)^41^ was obtained from the Protein Data Bank (www.rcsb.org)^71^ and parameterized according to the GROMOS96^72,73^ force field with the 54A7^74,75^ parameter set. A lipid membrane comprising dimiristoylphosphatidylcholine (DMPC) with 20% cholesterol (CHOL) was obtained from the Computational Molecular Biophysics Group at the Georg-August-Universität Göttingen (http://cmb.bio.uni-goettingen.de/cholmembranes.html)^76,77^. For DMPC, the parameterization developed by Poger *et al*.^78,79^ was used due to its ability to accurately reproduce properties of the lipid bilayers as area and volume per lipid ratios^78,80,81^, fluid-phase^78,79,82,83^ and solvation^78,79,83^ properties. Cholesterol and ATP parameterization was prepared online through the Automated Topology Builder (ATB) and Repository^84,85^ or PRODRG^86^ servers, manually curated and added with Merz-Kollman partial charges, assigned through *ab initio* calculations at the Hartree-Fock level of theory using the 6–31 G(d) basis set in Gaussian03^87^ program. Protein manipulation, protonation and homology modeling was performed in MOE 2015.1001^88^. The GROMACS simulation package 5.0.7^89–92^ was used for the MD simulations and protein insertion into the lipid membranes through the *g_membed* ^93^ module. Areas per lipid (*A*_L_) and thickness (*D*_HH_) of all systems were calculated with the MembPlugin^62^ extension in VMD^94^. Principal Component analysis (PCA) for the evaluation of the protein’s motion patterns was performed using the ProDy^95,96^ software through the NMWiz plugin in VMD^94^. Free energies of binding were calculated using *g_mmpbsa* ^97^, with polar solvation energies corrected by generating ion-accessibility and dielectric maps incorporating the membrane environment (dielectric slab constant is set to 2.0 using the *draw_membrane2* program) through in-house python scripts^98^. Both VMD and MOE were used for molecular inspection and visualization.

### Systems Construction

Due to a higher identity and similarity between *ABCG5* and *ABCG2* (27% and 48% respectively and better than *ABCG8*), and to avoid homology errors that may arise from crystallographic asymmetries in the *ABCG5/G8* heterodimer, one of the initial steps was the assembly of an *ABCG5* homodimer by superimposing a copy of the *ABCG5* (chain C) structure on the *ABCG8* transporter (chain D), being chain C chosen over chain A due to its higher quality (as described in the Full wwPDB X-ray Structure Validation Report^99^, available at http://ftp.wwpdb.org/pub/pdb/validation_reports/do/5do7/ 5do7_full_validation.pdf).

### Missing sequences

In order to obtain a full-length *ABCG5*, three sequences that were missing in the initial crystallographic structure were built: the A-loop (residues 47–66), the linker (residues 349–354) and the long extracellular loop (ECL, residues 590–598), based on the Chou-Fassman Secondary Structure Prediction server^100^. While the linker and ECL sequences were assessed as disordered, the A-loop was predicted to be a beta hairpin^101^, similar to that found in P-gp. Therefore, this segment was constructed using the *Protein Builder* module in MOE, followed by protonation and energy minimization, keeping the distance between the amine and carboxyl groups to the experimental one found in the crystallographic structure (4.5 Å). The resulting structure was then solvated, neutralized by adding an adequate number of counter ions and further studied in GROMACS through a series of eight MD simulations, each one with 50 ns duration and initial random velocities assigned from the temperature-related Maxwell-Boltzmann distribution. All obtained structures were evaluated regarding the final partial energies of the system, Ramachandran^102^ plots, and visual inspection. The most suitable conformation was added to the *ABCG5* crystallographic structure to be used as a template for the *ABCG2* homology model generation. The remaining sequences (linker and ECL) were modeled as coils.

### Homology modeling

To minimize possible clashes between atoms of the crystallographic structure and the added missing peptide sequences, prior to homology modeling a short energy minimization step took place while keeping the crystallographic structures’ heavy atoms spatially restrained. After loading the *ABCG2* fasta sequence (retrieved from UniProt, accession number Q9UNQ0), a homology model was built in MOE (*Homology Model* module), creating 100 mainchain models with 10 sidechain samplings at 300 K, in a total of 1000 models (Amber:EHT force-field). All other options were set as default. However, visual evaluation of the final model detected several problems, mainly related to mismodeled helical domains. Therefore, the secondary structure of these domains was predicted in CFSSP and built *de novo* in the homology model: while TM helices 4 and 5 were partially rebuilt as fully helical only from the distorted portions up to the nearest loop, the connecting helical domains following the NPXDF motif (two α-helices that precede the coil linking the NDB to the TMD, named CnH in *ABCG5/G8* and C2a/C2b in hABCG2) were completely rebuilt as fully helical, keeping its relative location similar to the original spatial coordinates in the *ABCG5* experimental structure.

### Linker equilibration

Since the linker connecting the NBD-TMD (residues 354–375) is longer than in *ABCG5*, we performed an additional computational study of this structure in GROMACS in the presence of a DMPC membrane. To that matter, after building the coil in MOE, a system comprising residues 332–389 (helix-linker-helix) was ported into GROMACS, inserted in an adequate membrane patch, solvated and neutralized with an adequate number of counter ions. Then, the system was energy minimized and equilibrated through a *NVT* run (10 ps, 303 K), followed by a short 4 ns *NpT* run (for membrane equilibration, 1 bar) and a 50 ns *NpT* run while keeping the flanking helices 332–353 (cytosolic) and 374–389 (anchored at the membrane interface) spatially restrained to allow the equilibration of the connecting coil. At the end, the system was evaluated and the most favorable conformation was inserted into the *ABCG2* homology model. The quality of the model was assessed through online validation servers as ERRAT^103^, MolProbity^104,105^, PROCHECK^106,107^ and SwissModel Structure assessment^108–110^ tools.

### Construction of membrane systems and refinement protocol

The relative position of the lipid bilayer was taken from the OPM^111^ database. Accordingly, a DMPC membrane with 20 molar-% cholesterol was chosen because i) cholesterol is required for optimal *ABCG2* activity^27^ probably acting as an allosteric regulator^112^, ii) in the original publication the protein was also reconstituted in DMPC bicelles in the presence of cholesterol prior to the crystallization step and ii) it provides an optimal hydrophobic environment to accommodate the hydrophobic thickness for the *ABCG2* transmembrane domains (PPM server prediction:^113^ 28.6 ± 0.9 Å). After membrane insertion, the system was water soaked (59.982 molecules) and neutralized with 22 chlorine ions, originating a system with 221.728 atoms. The system was then energy minimized and a 10 ps *NVT* run at 303 K followed, above the DMPC gel-fluid phase transition^114,115^. Then, the DMPC:CHOL membrane was allowed to correctly adjust to the protein structure through a 20 ns *NpT* run where all the protein’s heavy atoms were kept restrained. Finally, in order to better equilibrate all the *de novo* modeled domains, a refinement protocol was applied by progressively removing the heavy atoms’ spatial restrictions through a series of three sequential 10 ns *NpT* runs for the modeled helices (residues 310–354), linker (residues 355–375) and both helix-linker (residues 310–375) respectively. The resulting system was the starting point for a 200 ns unrestrained *NpT* run. Two additional systems were obtained by removing one DMPC molecule, found to be located between both *ABCG2* monomers, at the beginning (200 ns MD run) or after 100 ns of the 200 ns *NpT* run (for another 100 ns), for a total of 500 ns simulation time. At the end, the quality of all three models was again assessed by the previously used validation servers.

### ATP systems

Using the final conformation of the 200 ns *NpT* run and to assess the effect of ATP binding on the *ABCG2* homodimer, two ATP-Mg^2+^ complexes were placed in the nucleotide-binding domains (one for each monomer), with the magnesium ion in the vicinity of D210 (Mg^2+^-chelating residue), the ribose moiety close to the Walker A motif and the adenine next to the A-loop, followed by a short energy minimization step in MOE to allow the residues sidechains to adjust to the presence of ATP. Then, in GROMACS, the protein was further allowed to equilibrate to the presence of the nucleotide through a 10 ns *NpT* run where ATP-Mg^2+^ coordinates were kept spatially restrained. Afterwards, a 100 ns unrestrained *NpT* run was performed.

### Simulation parameters

For all simulations, periodic boundary conditions (PBC) were applied. Simple energy minimizations were performed using the steepest descent method. All *NVT* runs were performed at 303 K using a Velocity-rescale (V-rescale)^116^ thermostat. *NpT* runs used the Nosé-Hoover^117,118^ thermostat and the Parrinello-Rahman^117,119^ barostat for temperature (303 K) and pressure (1 bar) coupling, respectively. Due to the presence of membranes, pressure equilibration was achieved through a semi-isotropic pressure coupling, with the systems’ compressibility defined as 4.5 × 10^−5^ bar^−1^ and the initial box was defined with dimensions *xyz* of 17.37 × 11.58 × 13.50 nm^3^. The Particle Mesh Ewald (PME) with cubic interpolation^120,121^ was employed, with cut-off radius of 12 Å for both electrostatic and van der Waals interactions and an FFT grid spacing of 0.16 nm for long-range electrostatics. Group-based or Verlet^122^ cut-off schemes were applied for the calculation of non-bonded interactions done on CPU or GPU respectively. The SETTLE^123^ (for water molecules) or LINCS^124,125^ algorithms were used to constrain all bond lengths.

### Molecular Docking

Substrates (N = 69) and modulators (N = 19) were selected according to the publication by Mo *et al*.^10^ Molecular docking was made with all three *ABCG2* homology models obtained at the end of the MD simulations. MarvinSketch v17.2.20^126^ was used for drawing and minimizing structures. PDBQT files were generated with AutoDockTools^127^ for further utilization in AutoDock VINA v1.1.2^128,129^ docking software. The binding location was defined by a docking box including the whole transmembrane domains, with dimensions *xyz* of 16.87 × 14.06 × 16.87 Å^3^ (*xy* corresponds to the membrane plane). Due to the large search space volume (over 27.000 Å^3^), ‘exhaustiveness’ parameter was manually set to 50. Visual inspection of the docking poses was made in MOE to allow the identification of individual docking zones. For each site, the docked molecules were overlapped with the cavity search results by EPOS^BP^ ^130,131^, thus allowing the identification of lining atoms (within a distance of 5 Å from the pocket probes), mean pocket volumes and polarities (ratio of the sum of N, O, and S atoms to the sum of N, O, S, and C atoms). Graphical images of the docking poses and molecular maps of the pocket surfaces were generated in MOE.

### Data availability

The final configuration of the MD refined *ABCG2* homology structure and the ATP systems are available for download at our website (http://chemistrybits.com/).
