# Author Correction: Structure-function relationships in ABCG2: insights from molecular dynamics simulations and molecular docking studies

**DOI:** 10.1038/s41598-018-24602-w

**Published:** 2018-04-17

**Authors:** Ricardo J. Ferreira, Cátia A. Bonito, M. Natália D. S. Cordeiro, Maria-José U. Ferreira, Daniel J. V. A. dos Santos

**Affiliations:** 1 Research Institute for Medicines (iMed.ULisboa), Faculty of Pharmacy, Universidade de Lisboa, Av. Prof. Gama Pinto, 1649–003 Lisboa, Portugal; 2 LAQV@REQUIMTE, Department of Chemistry & Biochemistry, Faculty of Sciences, University of Porto, Rua do Campo Alegre, 4169–007 Porto Portugal

Correction to: *Scientific Reports* 10.1038/s41598-017-15452-z, published online 14 November 2017

This Article contains typographical errors in the Acknowledgements section.

“This project received funding from European Structural & Investment Funds through the COMPETE Programme and from National Funds through FCT – Fundação para a Ciência e a Tecnologia under the Programme grants PTDC/QEQMED/0905/2012, UID/DTP/04138/2013 and SAICTPAC/0019/2015 (FFUL). Ricardo J. Ferreira acknowledges the Ph.D grant from FCT, SFRH/BD/84285/2012. This work additionally received financial support from FCT through national funds, and co-financed by the European Union (FEDER funds) under the Partnership Agreement PT2020, through projects UID/QUI/50006/2013 and POCI/01/0145/FEDER/007265 (LAQV@REQUIMTE).”

should read:

“This work received financial support from the European Union (FEDER funds POCI/01/0145/FEDER/007265) and National Funds (FCT/MEC, Fundação para a Ciência e Tecnologia and Ministério da Educação e Ciência) under the Partnership Agreement PT2020 UID/QUI/50006/2013. Ricardo J. Ferreira acknowledges the PhD grant from FCT, SFRH/BD/84285/2012. This project also additionally received funding from European Structural & Investment Funds through the COMPETE Programme and from National Funds through FCT – Fundação para a Ciência e a Tecnologia under the Programme grants PTDC/QEQMED/0905/2012.”

